# *Polychromophilus* spp. (Haemosporida: Plasmodiidae): First Molecular Detection in Bat Flies From Brazilian Bats

**DOI:** 10.1111/1749-4877.13001

**Published:** 2025-05-29

**Authors:** Bruno S. Mathias, Vinicio R. De Lima, Gustavo Graciolli, Nubia R. M. F. Rocha, Jaciara O. J. Costa, Herbert S. Soares, Arlei Marcili, Karin Kirchgatter

**Affiliations:** ^1^ Programa de Pós‐Graduação em Medicina Tropical, Faculdade de Medicina Universidade de São Paulo São Paulo Brazil; ^2^ Universidade Federal de Mato Grosso do Sul Campo Grande Brazil; ^3^ Laboratório de Bioquímica e Biologia Molecular Instituto Pasteur São Paulo Brazil; ^4^ Departamento de Medicina Veterinária Preventiva e Saúde Animal Universidade de São Paulo São Paulo Brazil; ^5^ Programa de Pós‐Graduação em Saúde Única Universidade Santo Amaro São Paulo Brazil

**Keywords:** Atlantic Forest, bat, bat fly, Brazil, haemosporidian, *Polychromophilus*

## Abstract

Haemosporidian parasites exhibit a wide range of vertebrate hosts and corresponding insect vectors. Among mammals, bats host the most diverse array of haemosporidians, with seven genera identified. The genus *Polychromophilus* is exclusive to bats and is globally linked with hematophagous flies of the genera *Basilia*, *Nycteribia*, and *Penicillidia* as potential vectors. In Brazil, recent molecular studies have detected *Polychromophilus* in bats from the Cerrado and Atlantic Forest biomes; however, its vectors in the country remained unidentified. This study analyzed the haemosporidians infection of bat flies (24 Nycteribiidae and 43 Streblidae) collected from 13 bat species in the Legado das Águas. The bat–fly associations revealed highly specialized interactions, particularly among *Basilia* flies and *Myotis* bats. Notably, a rare interaction between *Megistopoda proxima* and *Carollia perspicillata* was also observed. Two specimens (3%) of nycteribiid flies (*Basilia speiseri* and *Basilia lindolphoi*), both collected from *Myotis nigricans*, tested positive for infection with *Polychromophilus* spp. Using *cytb* gene sequences, we examined the phylogenetic relationships of these *Polychromophilus* lineages with other global lineages. We identified two haplotypes, each clustering in distinct clades within the *Polychromophilus murinus* group. The presence of these parasites was further confirmed by sequencing of the *clpc* gene from the apicoplast genome and the nuclear *asl* gene. This study represents the first molecular detection of *Polychromophilus* spp. in a vector in Brazil, 50 years after its morphological description in the salivary glands of *Basilia*. These findings provide novel insights into the ecological networks in host–parasite–vector interactions in a preserved neotropical environment.

## Graphical abstract

This study identifies Polychromophilus sp. in blood‐feeding flies (*Basilia speiseri* and *Basilia lindolphoi*) from *Myotis nigricans* bats in Brazil, marking the first molecular detection of the parasite in an insect vector in this country. Two haplotypes were identified, both within the *Polychromophilus murinus* group. These findings confirm *Basilia* flies as vectors of *Polychromophilus*, highlighting the wide distribution of this haemosporidian pathogen in bat populations.

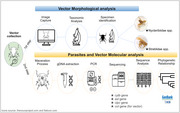

## 1 Introduction

Brazil is recognized for its vast diversity of mammals, with the order Chiroptera (bats) playing a prominent role. Comprising nine families, 68 genera, and 182 species, bats hold the second position in species richness among mammals in the country, only behind rodents (Rodentia), which have 235 species. The bat families in Brazil include Emballonuridae (17 species), Phyllostomidae (94), Mormoopidae (4), Noctilionidae (2), Furipteridae (1), Thyropteridae (5), Natalidae (1), Molossidae (32), and Vespertilionidae (26) (Nogueira et al. 2014; Peracchi et al. 2006). These bats are distributed throughout the national territory, inhabiting all Brazilian biomes (Amazon, Cerrado, Caatinga, Atlantic Forest, Pantanal, and Pampas), as well as urban areas within fragments of these biomes (Dias et al. 2013; Nogueira et al. 2014; Paglia et al. 2012; Peracchi et al. 2006; Reis et al. 2013, 2017; Simmons 2005).

Bats occupy a wide range of habitats for shelter and rest, such as caves, rock crevices, tree hollows, palm leaves, fallen logs, and river roots, and they also adapt to urban environments. This ecological flexibility allows some bat species to serve as natural reservoirs for parasites, facilitating their maintenance and dissemination (FAO 2011; Reis 1981; Reis et al. 2008, 2013). Among the parasites associated with bats are viruses, bacteria, fungi (FAO 2011; de Oliveira Corrêa et al. 2013; França and Langoni 2025), and protozoa, including haemosporidians like *Polychromophilus* (Ceballos‐Pérez et al. 2024; Pacheco and Escalante 2023; Timm et al. 2025).

Protozoa from the phylum Apicomplexa, order Haemosporida, also known as haemosporidians, are notable for their diverse vertebrate hosts (amphibians, reptiles, birds, and mammals) and associated insect vectors (Ceballos‐Pérez et al. 2024; Guillén‐Rodríguez et al. 2024; Harl et al. 2025; Martinsen et al. 2008; Schaer et al. 2013; Timm et al. 2025; Valkiūnas 2005). Among mammals, bats stand out for hosting the greatest diversity of haemosporidians, with around nine genera described for this group (Martinsen et al. 2008; Valkiūnas 2005). In addition to the genera *Plasmodium* (associated with malaria) and *Hepatocystis* (specific to mammals), five genera infect bats exclusively: *Polychromophilus* (including *Bioccala* and *Biguetiella*), *Nycteria*, *Dionisia*, *Johnsprentia*, and *Sprattiella* (Landau et al. 2012a, 2012b; Pacheco and Escalante 2023; Perkins and Schaer 2016). In bats, *Plasmodium*, *Hepatocystis*, and *Polychromophilus* are associated with blood‐feeding dipterans from the families Culicidae, Ceratopogonidae, and Nycteribiidae, respectively, while the vectors for other genera remain unknown (Galen et al. 2018; Ramasindrazana et al. 2018; Schaer et al. 2013, 2015; Witsenburg et al. 2012). It is worth mentioning, although *Polychromophilus* is primarily associated with flies from the Nycteribiidae family (Bajić et al. 2023; Megali et al. 2011; Obame‐Nkoghe et al. 2016; Ramasindrazana et al. 2018; Rosyadi et al. 2022; Sándor et al. 2021; Szentiványi et al. 2020; Timm et al. 2025; Witsenburg et al. 2015), a study conducted in Gabon detected DNA from this genus in bat flies from the Streblidae family (Obame‐Nkoghe et al. 2016).

The parasite–vector relationship is well established, including at the level of the bat host family. Bats from the family Vespertilionidae are commonly parasitized by haemosporidians of the genus *Polychromophilus*, which are associated with blood‐feeding flies from the family Nycteribiidae. These flies have coevolved with their hosts, becoming exclusive to the Chiroptera order (Gardner 1988; Dick and Patterson 2006; Graciolli et al. 2007; Dick and Dittmar 2014; Ramasindrazana et al. 2018; Szentiványi et al. 2020; Bajić et al. 2023).

Flies of the genus *Basilia*, belonging to the family Nycteribiidae, are of particular interest due to their high species diversity, with over 100 described globally, 50 of which are exclusive to the Americas (Graciolli and Dick 2023; Guimarães 1966). The genus *Basilia* shows a clear preference for bats of the family Vespertilionidae (Graciolli 2004; Graciolli and Dick 2023; Guimarães 1966), and previous studies have identified this genus as a potential vector of *Polychromophilus* in bats from Northeastern Brazil (Garnham et al. 1971).

Although studies in Brazil on hematophagous flies have focused on morphological and taxonomic aspects, as well as specific associations with bats (Barbier and Bernard 2023; Graciolli 2004; Graciolli and Dick 2023; Reis et al. 2017), there are currently no molecular records of *Polychromophilus* in bat flies since the pioneering work conducted in 1971 (Garnham et al. 1971). This study aims to fill this gap by reporting for the first time the molecular detection of *Polychromophilus* spp. in bat flies from the family Nycteribiidae collected from Brazilian bats. Molecular detection of these parasites contributes to the knowledge of haemosporidian distribution in bats and the understanding of ecological interactions between blood‐feeding vectors and their mammalian hosts.

## 2 Materials and Methods

### 2.1 Ethics Statement

All specimens were collected and handled in accordance with the necessary authorizations issued by the Brazilian government. The project was approved by SISBIO (System for Authorization and Information on Biodiversity) and ICMBio/MMA (Chico Mendes Institute for Biodiversity Conservation/Ministry of the Environment), under authorization number 58632‐1.

### 2.2 Sampling

**FIGURE 1:**
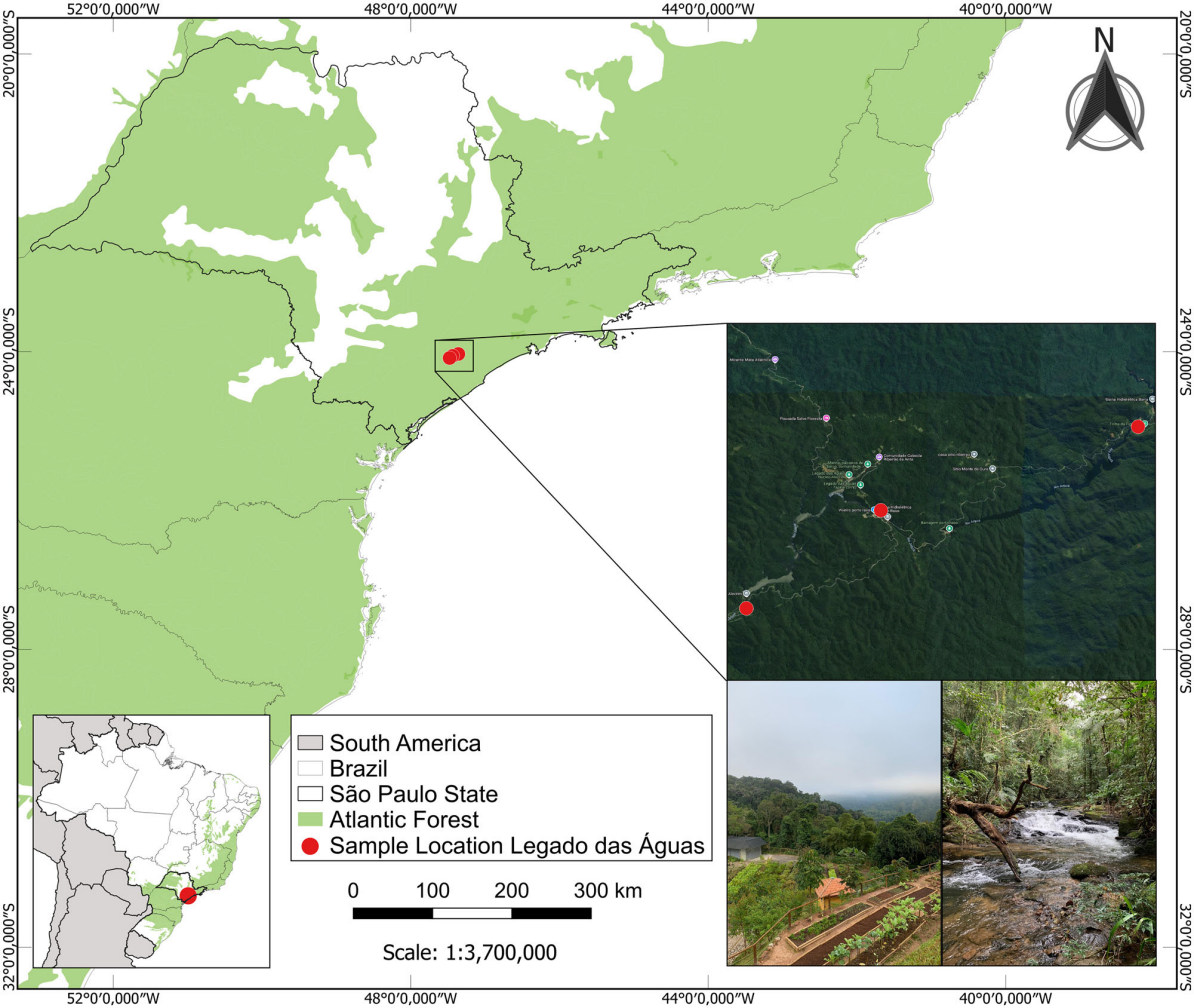
Map of the collection points for hematophagous flies of the Legado das Águas within a fragment of the Atlantic Forest. Photos Herbert S. Soares.

Bats were captured from 6 to 10 p.m., for 5 nights in 3 campaigns, using mist nets and, in some cases, through active searches in shelters, then transported in cloth bags to a field laboratory. Identification was performed based on taxonomic keys and original descriptions (Emmons and Feer 1990; Vizotto and Taddei 1973). Some specimens whose species identification was not possible in the field were euthanized through sedation with xylazine (1 mg/kg) and ketamine (10 mg/kg), followed by inhalation of isoflurane, and subsequently preserved in 10% formaldehyde for future identification and storage at the Museum of Zoology of the University of São Paulo. In addition to the animals whose identification was not possible in the field, one individual of each captured species was also euthanized, following standardization and authorization from SISBIO/ICMBio, for deposition as a voucher specimen due to the lack of previous descriptions of the bat species occurring in the reserve area. Hematophagous dipterans analyzed in this study were collected from these bats, stored in microtubes with 70% ethanol, and kept in a −20°C freezer. These specimens were obtained between 2018 and 2021 in the Legado das Águas reserve, the largest private Atlantic Forest reserve in Brazil. This reserve is located in the municipalities of Miracatu and Tapiraí, in the Vale do Ribeira region, 122 km south of São Paulo's capital, within the southern portion of the Serra do Mar ecological corridor. Covering 31,000 hectares, the Legado das Águas is continuously connected to other Conservation Units, forming a vital ecological corridor between coastal and inland areas. It represents the largest remaining continuous area of Atlantic Forest with minimal human intervention, a preservation state attributed to the region's low population density and limited economic development (Figure 1).

### 2.3 Morphological Analysis

All the dipterans received underwent a sorting process, where they were separated and individually identified in tubes containing 100% ethanol. This process was crucial, as some specimens belonged to the same individual (bat), which could compromise the data. To identify the organisms through physical characteristics, morphological and taxonomic analysis was conducted using a Leica digital camera, model DFC320, mounted on a Leica stereoscopic microscope, model S6. Photos were taken from at least three different angles, including ventral, dorsal, lateral views, and, in some cases, images of the insect's mouthparts, always with the respective scale as a measurement parameter. Bat fly species were identified using taxonomic keys (Guimarães and D'Andretta 1956; Graciolli 2004; Guerrero 2019), adhering to criteria that take into account the variation of morphological characters between sexes, since the presence of specimens of both sexes is crucial for correct taxonomic identification. Secondary sexual characteristics, such as the structure of the genital organs and certain parts of the body, such as the abdomen and wings (when present), may be essential to differentiate species of very similar flies.

### 2.4 DNA Extraction and Molecular Detection of Haemosporidian Parasites

The dipterans (whole specimen) were transferred to tubes containing 300 µL of phosphate‐buffered saline (PBS) and subsequently macerated using the FastPrep‐96 (MP Biomedicals, Solon, OH) in combination with two 1.4 mm ceramic beads (MagNA Lyser Green Beads—Roche Molecular Systems) coated with 6.35 mm zirconium oxide (MP Biomedicals). The maceration process was conducted for 3 to 6 min at a speed of 1800 rpm. Subsequently, the samples were centrifuged at room temperature for 5 to 10 min at 14,000 rpm.

DNA extraction was performed using the Unixtractor Whole Blood Genomic DNA Extraction Kit from Uniscience semi‐automated platform for nucleic acid extraction using magnetic beads and Wizard SV 96 Genomic DNA Purification System kit (PROMEGA, Madison, WI), according to the manufacturer's instructions with an additional wash. DNA was eluted in 100 µL of nuclease‐free water and stored at −20°C until analysis.

A fragment of approximately 1.1 kb (about 92% of the gene) from the mitochondrial *cytb* gene was amplified using nested polymerase chain reaction (PCR), with standard precautions taken to avoid cross‐contamination. The PCR was carried out following the method described (Perkins and Schall 2002), using primers DW2 and DW4 and 2 µL (50 ng) of genomic DNA in the initial reaction. For the nested reaction, 1 µL of the PCR product was used as a template, with primers DW1 and DW6.

For the *clpc* gene, a ∼500 bp fragment was amplified using nested PCR based on the method described (Martinsen et al. 2008). The initial PCR reaction used primers clpcF and clpcR, with 2 µL (50 ng) of genomic DNA. Subsequently, 1 µL of the initial PCR product was utilized as a template for a nested PCR with primers clpcF2 and clpcR2.

To amplify the *asl* gene, a fragment of approximately 240 bp was obtained using nested PCR, following the method described (Martinsen et al. 2008). The initial reaction used primers aslF and aslR, along with 2 µL (50 ng) of genomic DNA. A 1 µL aliquot of the initial PCR product was then used as a template in the nested PCR with primers aslF2 and aslR2.

PCR products were sequenced using the BigDye Terminator v3.1 Cycle Sequencing Kit on an ABI PRISM 3500 Genetic Analyzer (Applied Biosystems, Carlsbad, CA), employing nested PCR primers. For the *cytb* gene, sequencing was performed also with the oligonucleotides DW8 and DW3 (Perkins and Schall 2002). The sequences for *cytb*, *clpc*, and *asl* were then obtained and aligned with sequences available in the GenBank database.

### 2.5 Phylogenetic Analysis

The phylogenetic relationships among haemosporidian parasites were assessed using either partial sequences of the cytochrome b gene (*cytb*, 1116 bp) or a combined analysis of three genes (1462 bp): the mitochondrial cytochrome b gene (*cytb*, 725 bp), the nuclear adenylosuccinate lyase gene (*asl*, 206 bp), and the apicoplast caseinolytic protease C gene (*clpc*, 531 bp). Sequences obtained from the GenBank database were included in the phylogenetic trees and summarized in Table S1 (*cytb*) or Table S4 (multigene). Missing data for multigene analysis are shown as hyphens in Table S4 (*Polychromophilus* species without GenBank accession numbers in some columns). Sequence alignment was carried out using the ClustalW algorithm (Thompson et al. 1994) within MEGAX software (Kumar et al. 2018). Phylogenetic reconstruction was performed via Bayesian inference using MrBayes v3.2.0 (Huelsenbeck et al. 2001). MEGAX software (Kumar et al. 2018) was used to determine the optimal evolutionary model, with the GTR + G + I model selected based on Bayesian information criterion scores. Bayesian inference was conducted with two Markov Chain Monte Carlo runs of 8 million generations for *cytb* and 10 million generations for the concatenated analysis, sampling every 300 trees. After discarding the first 25% as burn‐in, the remaining 15 002 trees were used to construct a 50% majority‐rule consensus tree. The standard deviation of split frequencies was <0.01. The phylogenies were visualized and edited using FigTree version 1.4.0 (Rambaut 2010) and Inkscape v1.3.2 (Inkscape 2023).

### 2.6 Dipteran Species Molecular Identification

For specimens where morphological identification to the species level was not possible, a complementary analysis was performed using molecular analysis targeting the mitochondrial cytochrome c oxidase (COI) gene. A fragment of approximately 600 bp from the COI gene was amplified using the universal primers LCO1490 and HCO2198 (Folmer et al. 1994) following the PCR protocol based on Ruiz et al. (2010). The amplified fragments were directly sequenced using the corresponding flanking primers.

### 2.7 Bat and Bat Fly Interaction Network

To investigate the interactions between dipteran ectoparasites and bat species, we constructed an interaction network following the approach outlined by Dormann et al. (2009). Using the R programming language, we employed the libraries “bipartite” for constructing and visualizing bipartite networks, “vegan” for ecological community analysis, and “dplyr” for data manipulation and preparation. The network aim is to illustrate interaction patterns and reveal potential host specificity and community structure within this ecological system. The image file of the interaction network was edited in Canva (Canva Pty Ltd, Sydney, Australia), for the best presentation of the data.

Using the “bipartite” library, we have also measured: the H2’ index, consisting of a scale from 0 (completely generalistic interactions) to 1 (completely specialized interactions), quantifying the specialization of the parasitic interactions between ectoparasitic flies and bats; and the modularity (Q) of the interaction network, which ranges from −1 to 1 (values close to 0 indicate less separation between modules, values close to 1 indicate high modularization, and negative values suggest modularity lower than expected).

## 3 RESULTS

### 3.1 Fly Species Identification

We sampled 67 fly specimens of the following families and species: family Streblidae, *Anastrebla caudiferae* (three specimens), *Aspidoptera falcata* (seven specimens), *Megistopoda proxima* (six specimens), *Paraeuctenodes similis* (two specimens), *Strebla carvalhoi* (one specimen), *Strebla guajiro* (three specimens), *Strebla wiedemanni* (one specimen), *Trichobius dugesii* (one specimen), *Trichobius joblingi* (13 specimens), *Trichobius tiptoni* (six specimens); family Nycteribiidae, *Basilia lindolphoi* (one specimen), *Basilia* sp. (two specimens), *Basilia speiseri* (21 specimens). All bat fly specimens and their respective bat host species are listed in Table 1.

**TABLE 1:** Flies species examined for *Polychromophilus* detection in Legado das Águas, São Paulo, Brazil. Localities are shown in Table S2.

Host family	Host bat	Vector family	Flies species	Flies examined	Flies infected with haemosporidians (%)
Phyllostomidae	*Anoura caudifer*	Streblidae	*Anastrebla caudiferae*	3	0
			*Strebla carvalhoi*	1	0
			*Trichobius tiptoni*	5	0
	*Carollia perspicillata*		*Megistopoda proxima*	2	0
			*Paraeuctenodes similis*	2	0
			*Strebla guajiro*	3	0
			*Trichobius joblingi*	13	0
	*Desmodus rotundus*		*Strebla wiedemanni*	1	0
	*Glossophaga soricina*		*Trichobius dugesii*	1	0
			*Trichobius tiptoni*	1	0
	*Sturnira lilium*		*Aspidoptera falcata*	7	0
			*Megistopoda proxima*	4	0
Vespertilionidae	*Neoeptesicus* sp.	Nycteribiidae	*Basilia* sp.	2	0
	*Myotis nigricans*		*Basilia lindolphoi*	1	1 (100%)
			*Basilia speiseri*	21	1 (4.76%)
**TOTAL**				**67**	**2 (3%)**

We used DNA barcode to identify 08/67 bat fly specimens, previously identified by morphology only to the genus level: seven *Basilia* sp. (ID: 12_8,16_2, 22_1, 22_2, 62, 65_1, and 84) and one *Trichobius* sp. (ID: 66_2). Additionally, due to the absence of reference sequences of *B. speiseri* and *T. tiptoni* in the GenBank database, we also used six specimens of these species previously identified morphologically in this study (IDs: 12_5, 12_6, 12_7, 63_1, 130_1, and 130_2). The obtained sequences were used for comparison with our sequences of undefined species. As a result, the sequences of *Basilia* IDs: 12_8, 62, 65_1, and 84 showed 99% or 100% similarity with our reference sequences for *B. speiseri*, while sequence 16_2 showed 99% similarity with GenBank for *B. lindolphoi*. The specimens 22_1 and 22_2 remained as *Basilia* sp., showing approximately 94% similarity with both the database and our reference sequences. The sequence of *Trichobius* ID: 66_2 showed 100% similarity with our reference sequences. The bat fly species identified both morphologically and molecularly, along with their respective IDs and detailed information, are listed in Table S1. All sequences obtained were deposited in the GenBank database (PQ788868‐PQ788881).

### 3.2 Interaction Network

**FIGURE 2:**
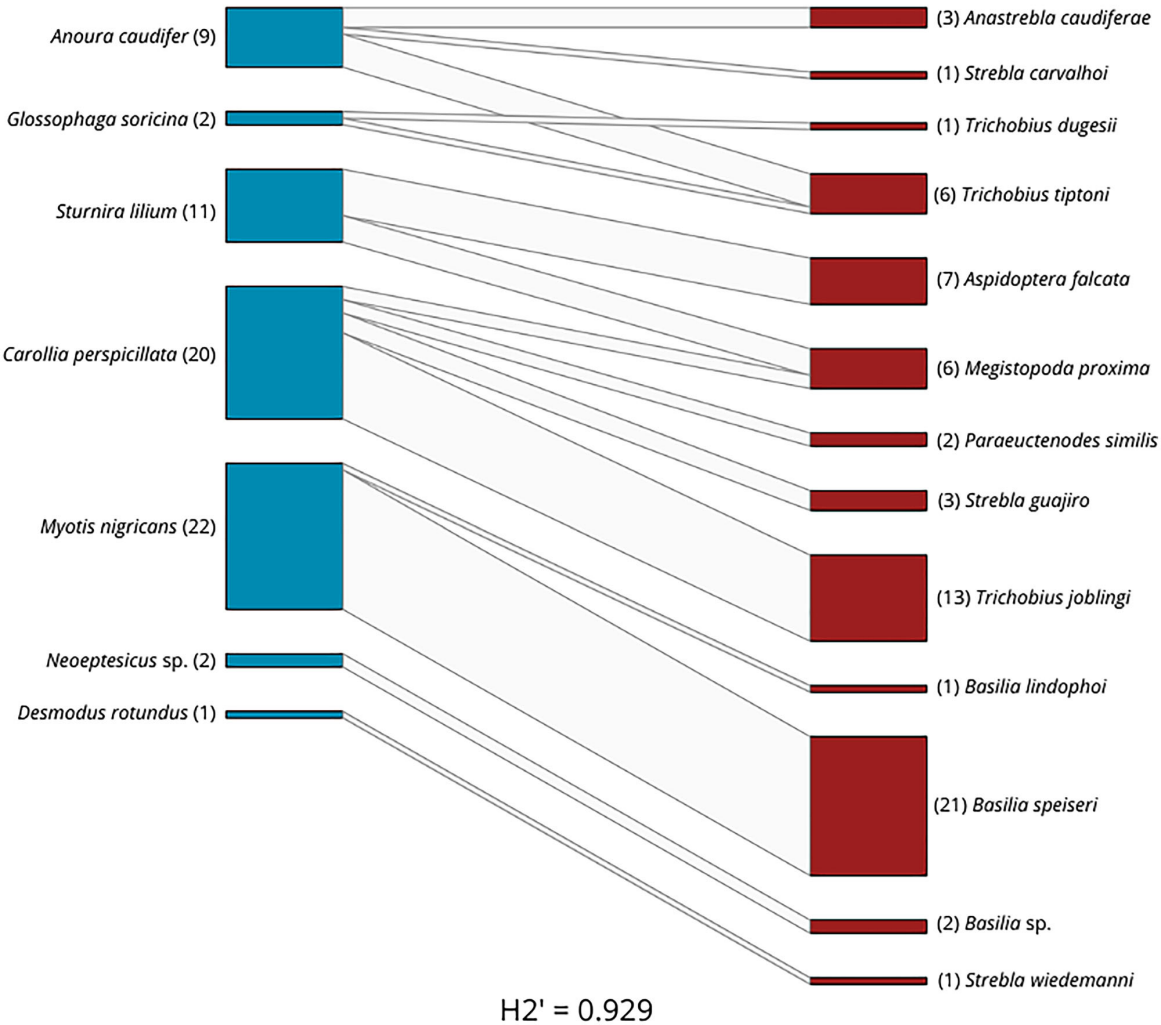
Interaction network between species of host bats and bat flies.

*Carollia perspicillata* was found to interact with four ectoparasite species: *M. proxima*, *P. similis*, *S. guajiro*, and *T. joblingi*. The interaction network reveals that *C. perspicillata* exhibited the highest number of interactions with different species, primarily with *T. joblingi* and some with *P. similis* and *S. guajiro*. Additionally, we found that the bat species *Myotis nigricans* interacted exclusively with flies from the genus *Basilia*, predominantly *B. speiseri* (Figure 2).

Among the bat fly species, *Trichobius* was the only genus interacting with three different bat species (*Glossophaga soricina*, *Anoura caudifer*, and *C. perspicillata*). *G. soricina* was observed in associations with two *Trichobius* species (*T. dugesii* and *T. tiptoni*) and *C. perspicillata* was the most generalist of the bats species observed in this study, showing interactions with four different species of flies (*M. proxima, P. similis, S. guajiro*, and *T. joblingi*). The bat family Vespertilionidae (specifically the specimens *M. nigricans* and *Neoeptesicus* sp.) and the bat fly family Nycteribiidae (with *Basilia* genera), interacting only between both families, evidencing the high specialization (H2’ = 0.929) and high modularity (*Q* = 0.72).

### 3.3 *Polychromophilus* Parasites and Phylogeny

The percentage of bat flies positive for *Polychromophilus* by PCR testing was 3%, with two out of 67 flies infected (Table 1). The positive samples were one male fly of the species *B. lindolphoi* (sample ID 16_2) and one female fly of the species *B. speiseri* (sample ID 85_1), both flies belonging to the family Nycteribiidae, collected from bats of the species *M. nigricans*, family Vespertilionidae, from the municipalities of Tapiraí and Miracatu in the state of São Paulo, respectively.

We analyzed the presence of single nucleotide polymorphisms (SNPs), genomic variants at a single base position, in the fragment of the mitochondrial *cytb* (1116 bp) sequences of *Polychromophilus* sp. isolates. Sequences obtained from Brazilian bat flies of the genus *Basilia* were compared with isolates from Brazilian bats and also with the more similar sequences from GenBank (#LN483038, *Polychromophilus* sp. obtained from *M. nigricans* collected in Panama; and #PP971136, *Polychromophilus deanei* from *Myotis albescens* from Colombia) (Table 2). Twenty‐two sites were polymorphic among the Brazilian sequences. The Colombian sequence (Ceballos‐Pérez et al. 2024) shared two nucleotides conserved among all the Brazilian sequences, while the Panamanian sequence showed two nucleic acid substitutions at the same positions, found only in this isolate (positions 247 and 512) (Table 2). *Polychromophilus* sp. isolates from bat flies presented one (16_2) or two (85_1) singleton SNPs (positions 25, 87, and 337), that is, SNPs that only show up once in a single individual (Table 2).

**TABLE 2:** Nucleic acid polymorphism in the mitochondrial cytochrome b (*cytb*) gene sequences of *Polychromophilus* sp. isolates from bat flies (16_2 and 85_1) and bats from Brazil, and bats from Panama and Colombia (MYOPA01 and PP971136).

Species	Genbank accession number	Host	25	87	126	219	246	247	261	273	297	334	337	339	405	423	512	603	789	792	810	811	853	885	945	1086
*Polychromophilus* sp.	OQ957065	*Myotis* sp.	G	A	T	C	C	T	A	T	T	T	T	A	T	T	T	G	T	C	C	T	C	A	T	A
*Polychromophilus* sp.	MW984518	*Myotis ruber*	G	A	A	C	T	T	A	T	T	T	T	T	T	A	T	A	T	C	C	T	C	A	T	A
*Polychromophilus* sp.	MW984519	*Myotis riparius*	G	A	A	C	T	T	A	T	T	T	T	G	T	A	T	A	T	T	C	T	C	A	T	G
*Polychromophilus* sp.	MW984520	*Myotis riparius*	G	A	A	C	T	T	A	T	T	T	T	G	T	A	T	A	T	T	C	T	C	A	T	G
*Polychromophilus* sp.	MW984522	*Myotis riparius*	G	A	A	C	T	T	A	T	T	T	T	T	T	A	T	A	T	C	C	T	C	A	C	A
*Polychromophilus* sp.	OQ957066	*Myotis* sp.	G	A	A	C	T	T	A	T	T	T	T	A	T	A	T	A	T	C	C	T	C	A	T	A
*Polychromophilus* sp.	OP503502	*Myotis riparius*	G	A	A	C	T	T	A	T	T	C	T	T	T	A	T	A	T	C	C	T	C	A	T	A
*Polychromophilus* sp.	MW98452	*Neoeptesicus diminutus*	G	A	A	T	T	T	C	A	T	T	T	A	C	A	T	A	C	T	T	C	T	T	T	A
*Polychromophilus* sp.	OQ957064	*Myotis ruber*	G	A	A	T	T	T	T	A	C	T	T	A	C	A	T	A	T	T	T	C	T	T	T	A
*Polychromophilus* sp.	PQ789623	*Basilia lindolphoi* (*Myotis nigricans*)	G	A	A	C	T	T	A	T	T	T	A	T	T	A	T	A	T	C	C	T	C	A	T	A
*Polychromophilus* sp.	PQ789624	*Basilia speiseri* *(Myotis nigricans)*	A	C	T	C	C	T	A	T	T	T	T	A	T	T	T	A	T	C	C	T	C	A	T	A
*Polychromophilus deanei*	PP971136	*Myotis albescens*	−	−	−	−	T	T	A	T	T	T	T	T	T	A	T	A	−	−	−	−	−	−	−	−
*Polychromophilus* sp.	MYOPA01	*Myotis nigricans*	G	A	A	C	T	C	A	T	T	T	T	T	T	A	G	−	−	−	−	−	−	−	−	−

The sequence obtained from bat fly 85_1 was the most divergent, showing 98%–99% identity with the other Brazilian sequences (with 8 or 15 nucleic acid substitutions) (Table S3). The Colombian sequence showed few substitutions (with 3 or none) and 99% identity, while the Panamanian sequence presented two to eight nucleic acid substitutions compared to the Brazilian bat fly sequences (98%–99% identity).

**FIGURE 3:**
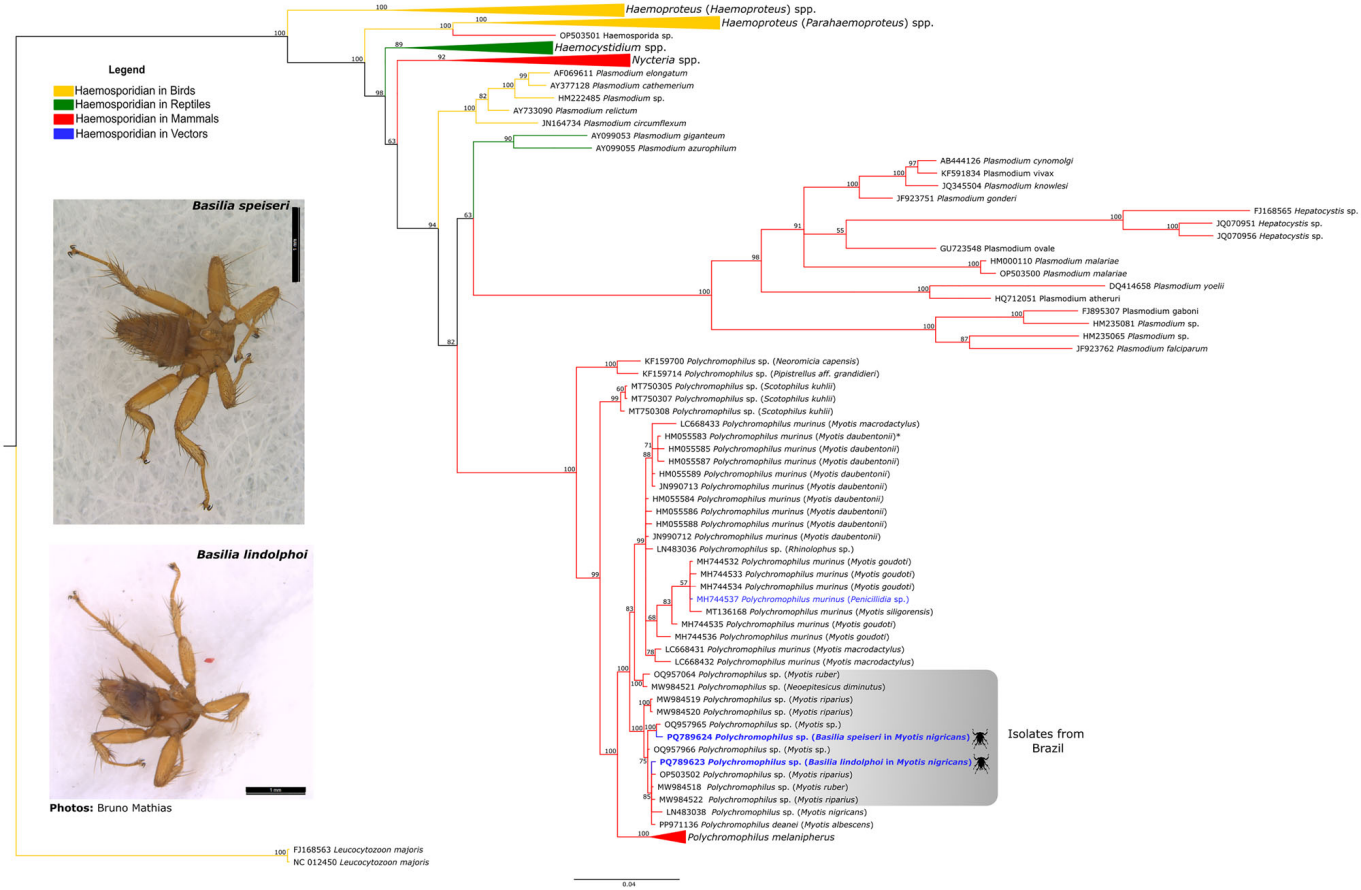
The Bayesian phylogeny, derived from the mitochondrial cytochrome b gene (*cytb*), incorporates 186 sequences that include both haemosporidian parasites from this study and reference sequences, aligned over 1116 bp (Table S1). *Leucocytozoon* spp. served as the outgroup. The support values for the nodes, represented as percentages, indicate posterior probabilities. The haemosporidian sequences found in mammals are highlighted by red branches, those from birds by yellow branches, those from reptiles by green branches, and those from vectors by blue branches. Sequences identified in this study are presented in blue bold. Additionally, sequence HM055583* has been documented in *Polychromophilus murinus* from *Eptesicus serotinus*, *Nyctalus noctula*, and *Myotis myotis*.

The phylogenetic reconstruction targeted the *cytb* gene and was generated using sequences available in the GenBank database, covering different genera of haemosporidians from various host species (Table S1). All *Polychromophilus* sequences found in this study and the sequences of the genus available in the GenBank database (Table S1) were included. This analysis did not produce conflicts in any of the major clades. Major genera and subgenera were recovered and grouped in the phylogenetic reconstruction into separate monophyletic clades (Figure 3). Except for the clade of the outgroup *Leucocytozoon*, seven clades are shown within the order Haemosporida. The *Polychromophilus* isolates from bats and vectors in different zoogeographic regions cluster into a monophyletic clade (posterior probability of 100), consisting of six subclades (with posterior probabilities >99), with *Polychromophilus* found in bat flies segregated into two of them (Figure 3).

The phylogenetic analysis positioned *Polychromophilus* as a sister clade to a group containing *Plasmodium* species that infect reptiles and mammals, as well as *Hepatocystis* species found in primates and rodents. All *Polychromophilus* sequences from bats worldwide formed a monophyletic clade with a high posterior probability of 100, consisting of eight subclades. Interestingly, all *Polychromophilus* sequences from Brazilian bat flies and bats were grouped within a single one of these subclades (Figure 3).

The first subclade includes parasite sequences from vespertilionid bats in Guinea, such as *Pipistrellus aff. grandidieri* (KF159700) and *Neoromicia capensis* (KF159714) (Figure 3). The second, separate subclade contains *Polychromophilus* sequences from *Scotophilus kuhlii*, a vespertilionid species from Thailand (MT750305, MT750307, and MT750308), forming this group (Figure 3). The third *Polychromophilus* subclade, with a posterior probability of 98, consists of sequences from *Polychromophilus murinus* obtained from bats in Europe (Switzerland and Bulgaria), Madagascar, Thailand, and Japan. This clade exclusively includes a *Polychromophilus* isolate, likely of the “*murinus* type,” from a *Rhinolophus* sp. bat in the family Rhinolophidae, which is not associated with either Vespertilionidae or Miniopteridae (Figure 3). The fourth subclade comprises sequences obtained from *Neoeptesicus diminutus* (MW984521) and *Myotis ruber* (OQ957064) in Brazil, exhibiting a posterior probability of 100. The fifth subclade comprises the *Polychromophilus* sequences obtained from *Myotis riparius* (MW984519 and MW984520) from Brazil. The sixth subclade grouped the *Polychromophilus* sequence from *Myotis* sp. bats (OQ957965 and OQ957966) along with the *B. speiseri* sequence collected from *M. nigricans* bats (85_1), also from Brazil. The seventh grouping of sequences consists of isolates from Brazilian *Myotis* bats (Mathias et al. 2023; Minozzo et al. 2021), the Brazilian bat fly *B. lindolphoi* collected from *M. nigricans* (16_2), *Polychromophilus* sp. isolates from *M. nigricans* in Panama, and *P. deanei* from *M. albescens* in Colombia. All *Polychromophilus melanipherus* sequences from bat flies and *Miniopterus* bat hosts were distinctly segregated into a separate subclade, highlighting a clear distinction between parasites of miniopterid and vespertilionid hosts (Figure 3).

The presence of *Polychromophilus* in Brazilian bat flies was confirmed by amplifying the *clpc* gene from the parasite's apicoplast in two samples (ID 85_1 and 16_2), which yielded fragments of approximately 500 bp. Additionally, the *asl* gene from the nuclear genome was amplified in sample ID 85_1, resulting in a fragment of 244 bp. When compared to sequences of the same target gene available on GenBank for the genus *Polychromophilus*, the *clpc* sequence from sample ID 85_1 exhibited 100% similarity with sequence OQ957063 from the Brazilian bat *Myotis* sp., followed by 99% similarity with other Brazilian sequences (OP503504 and OP503503) obtained from *M. riparius* (Mathias et al. 2023). In contrast, sample ID 16_2 showed 99% similarity with sequences OP503503, OQ957063, and OP503504. Furthermore, there was 96% similarity with the closest available sequences (LC715203 and LC715204) from *P. murinus*, described in Japanese bats of the species *Myotis macrodactylus* (Rosyadi et al. 2022).

**FIGURE 4:**
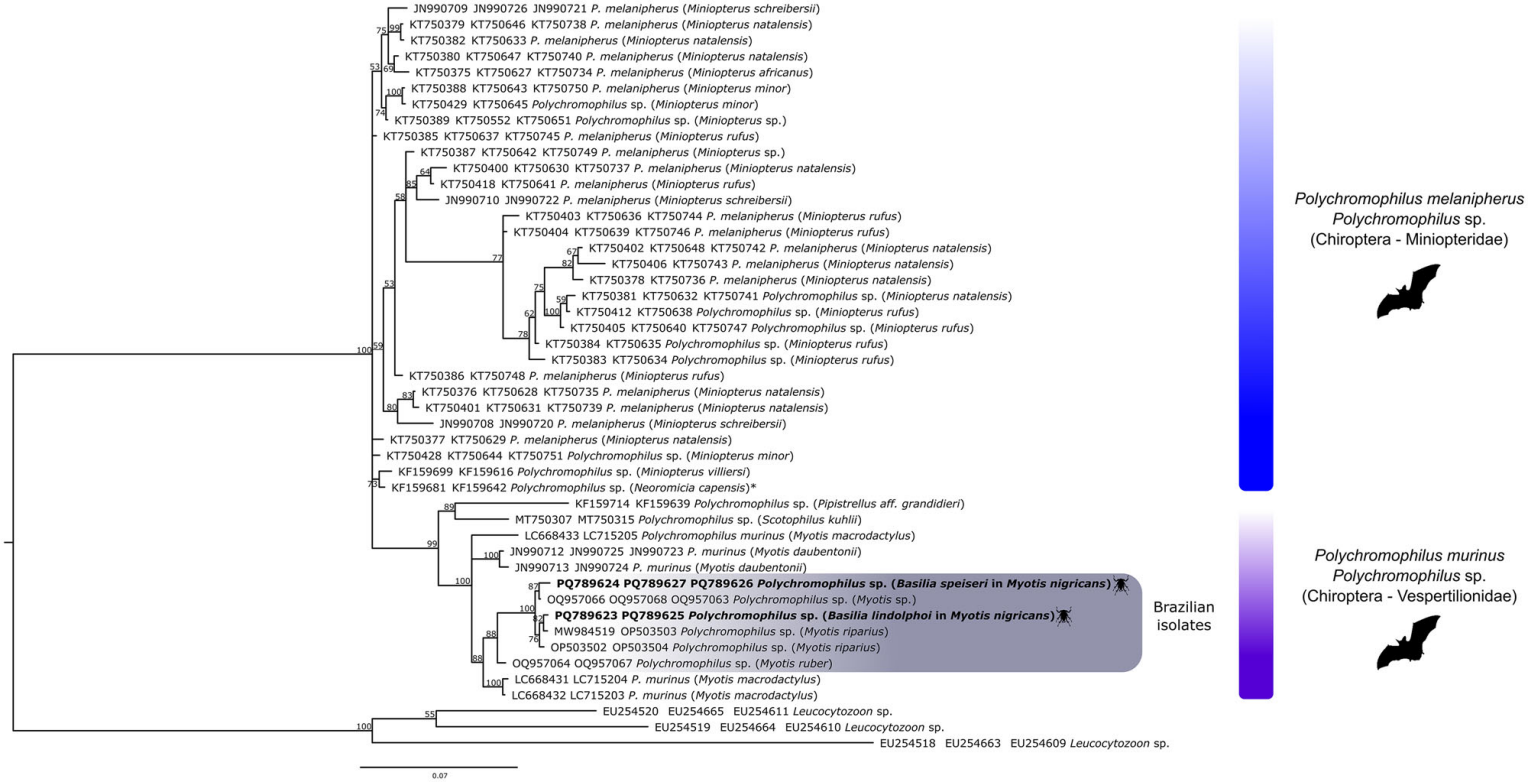
The Bayesian phylogeny was reconstructed based on the concatenated analysis of three genes: the mitochondrial cytochrome b gene (*cytb*, 725 bp), the nuclear adenylosuccinate lyase gene (*asl*, 206 bp), and the apicoplast caseinolytic protease C gene (*clpc*, 531 bp) from *Polychromophilus* spp. The sequences identified in this study are highlighted in bold, along with the reference sequences listed in (Table S4), totaling 47 sequences. The support values of the nodes (in percentage) represent posterior probabilities, and the Brazilian sequences are highlighted in gray boxes, while the sequences from bat flies are in bold. *Neoromicia capensis is a species of the vespertilionid group and is grouped with the miniopterids. *Leucocytozoon* spp. served as the outgroup.

A phylogenetic reconstruction was conducted using concatenated sequences from three genes: *cytb*, *asl*, and *clpc* (Figure 4). This analysis included the *Polychromophilus* sequences obtained in this study, along with all available sequences of this genus in the GenBank database (Table S4). The resulting phylogenetic tree topology confirmed the separation between parasites associated with miniopterid and vespertilionid hosts, with the exception of *N. capensis*, a vespertilionid species that grouped with miniopterid hosts. The vespertilionid subclade of *Polychromophilus* was further divided into three branches: one branch containing *P. murinus* sequences from Swiss bats (*Myotis daubentonii*), another branch with a single sequence from *M. macrodactylus* collected in Japan, and the third branch encompassing all *Polychromophilus* sequences found in Brazilian bats and bat flies, along with two additional sequences from *M. macrodactylus* from Japan (Rosyadi et al. 2022).

## 4 DISCUSSION

This study represents the first molecular detection of *Polychromophilus* spp. in blood‐feeding bat flies in Brazil, specifically in the species *B. speiseri* and *B. lindolphoi*, collected from *M. nigricans* bats in the Legado das Águas region, the largest continuous fragment of preserved Atlantic Forest in Brazil. The discovery is significant because, although previous studies have morphologically identified *Polychromophilus* in flies of the genus *Basilia* (Garnham et al. 1971), molecular detection in Brazilian bat flies has never been previously performed. Moreover, the new sequences obtained, in addition to confirming the presence of *Polychromophilus* in the invertebrate host, make it possible to infer phylogenetic relationships and possible species (Ceballos‐Pérez et al. 2024; Mathias et al. 2023; Timm et al. 2025).

The results show that 3% of the flies tested positive for *Polychromophilus*, a relatively low infection rate compared to other regions worldwide (Table 3). This prevalence may be influenced by ecological factors, such as bat population density and environmental conditions that affect the abundance of vectors and parasite transmission (Schaer et al. 2013). Additionally, sampling in a relatively preserved area of the Atlantic Forest directly impacts a higher concentration of bats and habitat stability, favoring the transmission cycle. This contrasts with more anthropized habitats, where fragmentation and vegetation reduction can decrease interactions between host and vector (Gottdenker et al. 2014; Keesing et al. 2010).

**TABLE 3:** Molecular detection of *Polychromophilus* spp. in bat flies worldwide and in this study.

Country or continent	Analysis specimens	Positive samples (positivity %)	Parasite species	Specimens positives	Host of positive specimens	Reference
Switzerland	7	1 (14.3%)	*Polychromophilus murinus*	*Nycteribia kolenatii*	*Myotis daubentonii*	Megali et al. 2011
^1^Europe	278	32 (11.5%)	*Polychromophilus melanipherus*	*Nycteribia schmidlii*	*Miniopterus schreibersii*	Witsenburg et al. 2015
^a^Gabon	1063	217 (20.7%)	*Polychromophilus melanipherus*	*Nycteribia schmidlii scotti, Penicillidia fulvida, Eucampsipoda africana, Raymondia huberi, Brachytarsina allaudi*	*Miniopterus inflatus, Rousettus aegyptiacus, Hipposideros caffer, Hipposideros gigas*	Obame‐Nkoghe et al. 2016
Madagascar	38	6 (15.7%)	*Polychromophilus murinus* *Polychromophilus melanipherus*	*Penicillidia* sp. (cf. *fulvida*), *Penicillidia leptothrinax, Nycteribia stylidiopsis*	*Miniopterus aelleni, Miniopterus manavi, Miniopterus gleni*	Ramasindrazana et al. 2018
South Africa and ^2^Europe	101	31 (30.7%)	*Polychromophilus melanipherus*	*Nycteribia schmidlii, Nycteribia schmidlii scotti*	*Miniopterus schreibersii, Miniopterus natalensis*	Szentiványi et al. 2020
Romania	53	23 (43.4%)	*Polychromophilus murinus* *Polychromophilus melanipherus*	*Polychromophilus conspicua*, *Nycteribia schmidlii, Polychromophilus dufourii, Nycteribia kolenatii, Nycteribia vexata*	*Miniopterus schreibersii, Myotis blythii, Myotis capaccinii, Myotis daubentonii, Myotis myotis*	Sándor et al. 2021
Japan	8	1 (12.5%)	*Polychromophilus melanipherus*	*Nycteribia* sp.	*Miniopterus fuliginosus*	Rosyadi et al. 2022
^3^Europe	215	33 (15.3%)	*Polychromophilus melanipherus*	*Polychromophilus conspicua, Polychromophilus dufourii; Nycteribia schmidlii*	*Miniopterus schreibersii, Rhinolophus ferrumequinum*	Bajić et al. 2023
^4^Europe	1131	287 (25.4%)	*Polychromophilus murinus* *Polychromophilus melanipherus*	*Basilia nana, Basilia nattereri, Nycteribia kolenatii, Polychromophilus dufourii*	*Myotis bechsteinii, Myotis blythii, Myotis capaccinii, Myotis crypticus, Myotis daubentonii, Myotis myotis, Myotis nattereri*	Timm et al. 2025
^a^Brazil	67	2 (3%)	*Polychromophilus* sp.	*Basilia lindolphoi, Basilia speiseri*	*Myotis nigricans*	This study

^1^ Croatia, Portugal, Spain, Italy, Slovakia, and France.

^2^ Hungary, Italy, Spain.

^3^ Serbia, Bosnia and Herzegovina.

^4^ Belgium, Bulgaria, France, Georgia, Hungary, Luxemburg, Russia, Spain, and Turkey.

^a^ These studies tested bat flies from the families Streblidae and Nycteribiidae, while the others tested only Nycteribiidae.

In studies conducted in Africa and Europe, infection rates of *Polychromophilus* in nycteribiid flies ranged between 11% and 43% (Table 3). This difference compared to the 3% observed in Brazil may be attributed to variations in collection methods, host and vector species sampled, and the ecological diversity of the studied habitats (Pacheco and Escalante 2023; Schaer et al. 2013). Interestingly, Timm et al. (2025) reported higher infection rates of *Polychromophilus* in nycteribiid flies of the genus *Penicillidia* compared to *Basilia*, suggesting that the larger body size of *Penicillidia* might offer a more favorable environment for parasite development and maintenance, potentially increasing infection likelihood. Although *Penicillidia* flies were not sampled in our study area, this hypothesis raises important questions about the role of vector morphology in parasite transmission dynamics and may partially explain differences in prevalence between regions. Furthermore, our detection of *Polychromophilus* in Brazil reveals considerable genetic diversity among the samples, with haplotypes clustering into distinct subclades within the *P*. murinus group. This finding suggests that bat parasite biodiversity in South America may be underestimated, particularly in highly diverse and preserved areas like the Atlantic Forest (Pacheco and Escalante 2023; Schaer et al. 2013). Future studies should include a broader diversity of nycteribiid genera and habitats to assess how morphological traits and ecological factors influence vector competence and parasite diversity, especially in neotropical regions where these interactions remain poorly understood.

Approximately 64% of the bat flies analyzed belong to the family Streblidae, which is primarily associated with bats of the family Phyllostomidae. All samples from this family tested negative for *Polychromophilus* spp., reinforcing the hypothesis that the transmission of these haemosporidians is more closely related to bat flies of the family Nycteribiidae, as previously evidenced (Bajić et al. 2023; Megali et al. 2011; Obame‐Nkoghe et al. 2016; Ramasindrazana et al. 2018; Rosyadi et al. 2022; Sándor et al. 2021; Szentiványi et al. 2020; Timm et al. 2025; Witsenburg et al. 2015). Obame‐Nkoghe et al. (2016) observed for the first time the relationship between Streblidae (two species of *Raymondia huberi* group, and *Brachytarsina allaudi*) and haemosporidians in bats (*Miniopterus inflatus*, *Rousettus aegyptiacus*, *Hipposideros caffer*, and *Hipposideros gigas*). However, none of the Streblidae or bat species mentioned above occur on the American continent (Dick and Miller 2010; Reis et al. 2017; Taylor and Turtle 2019), which limits the possibility of interaction described by Obame‐Nkoghe et al. (2016).

The generated interaction network highlights the high level of specialization between bats and their parasitic flies, with a high H2’ value and a high modularity value also, likely previous studies (Urbieta et al. 2021; Ramirez‐Martinez and Tlapaya‐Romero 2023). Consistent with previous studies (Bertola et al. 2005; López‐Rivera et al. 2024; Lourenço et al. 2020), the interaction between *Basilia* flies and bats from the *Myotis* genus is almost exclusive, with some exceptions, such as interactions with the bat genus *Neoeptesicus*, though specific identification to species level was hindered by poor sample conditions. Another rare yet intriguingly consistent finding was the interaction between *C. perspicillata* and *M. proxima*. Although *Megistopoda* flies are commonly associated with *Sturnira* bats, this study observed their interaction with two *Carollia* individuals, mirroring findings in the Atlantic Forest (Bertola et al. 2005) and more recently in Colombia (López‐Rivera et al. 2024). Despite the low numbers, this repeated association across different regions suggests that this interaction may be a recurrent phenomenon.

The phylogenetic analysis of the parasites indicates significant genetic diversity, with two distinct haplotypes clustered into different subclades within the *P*. murinus group. These haplotypes, when compared to sequences from other regions, such as Panama and Colombia (Borner et al. 2016; Ceballos‐Pérez et al. 2024), reveal new polymorphic sites unique to the Brazilian isolates. The presence of nucleotide polymorphisms in conserved regions of the mitochondrial gene (*cytb*) suggests that Brazilian parasites are subject to local selective pressures, supporting the idea that *Polychromophilus* populations in South American bats are genetically distinct, both within the group itself and compared to those from other regions (Pacheco and Escalante 2023; Schaer et al. 2013). Additionally, the clustering of some Brazilian sequences, including that from bat flies, with the Colombian sequence (Ceballos‐Pérez et al. 2024) suggests that these sequences may belong to the species *P. deanei*. However, future studies that implement the morphological analysis of parasitic forms are necessary for clarifying the taxonomy of this group.

The application of a multigene approach in this study provided a more robust understanding of the genetic diversity and evolutionary relationships within *Polychromophilus* spp. By analyzing multiple genetic markers, we could gain insights into lineage‐specific adaptations and potential host specificity, which are often missed in single‐gene analyses. This comprehensive approach strengthens the phylogenetic framework and supports more accurate evolutionary and ecological interpretations of parasite–host dynamics.

These findings are important for understanding the evolution of parasites in bats, as different species may harbor unique lineages of haemosporidians (Schaer et al. 2013). The clear distinction between bat parasites of the genus *Myotis* and other species, such as *Miniopterus*, observed in the phylogenetic analysis, supports the coevolution between these parasites and their specific hosts. This specificity may have significant implications for the distribution and transmission of parasites across different biomes and geographical zones (Gardner 1988; Schaer et al. 2013; Obame‐Nkoghe et al. 2016; Ramasindrazana et al. 2018; Sándor et al. 2021; Rosyadi et al. 2022).

Our results confirm the role of blood‐feeding flies of the genus *Basilia* as potential vectors of *Polychromophilus* (Garnham et al. 1971). The species *B. speiseri* (female) and *B. lindolphoi* (male) have been identified as key vectors associated with *M. nigricans*, confirming a previously described relationship (Garnham et al. 1971). The hematophagous behavior of both male and female Nycteribiidae, along with their high prevalence on Vespertilionidae bats (Dick and Patterson 2006) and their ability to easily move between bat hosts, make them an ideal vector for the *Polychromophilus*. The predominant presence of these flies in preserved environments, such as the Atlantic Forest, indicates that the dynamics of parasite transmission are linked to local ecological factors, including suitable habitats for bats and their flies (Barbier et al. 2024; Bertola et al. 2005; da Silva Biz et al. 2021; Dick and Patterson 2006).

In this context, anthropization, or the absence of it, should be considered a significant factor in interpreting the infection rates observed in this study. In preserved areas, the natural interactions between bats and their parasites tend to be more intense and stable, promoting a robust transmission cycle. Conversely, in highly anthropized environments, habitat fragmentation and anthropogenic pressure may reduce bat populations and their vectors, resulting in lower infection rates (Dick and Patterson 2006; Eriksson et al. 2023; Gottdenker et al. 2014; Keesing et al. 2010). Thus, anthropization appears to directly affect the ecological dynamics of the parasite, suggesting that both the absence and the excess of environmental alterations can influence the ecology of *Polychromophilus* and its vectors (Gottdenker et al. 2014).

The detection of *Polychromophilus* spp. in bat flies associated with *M. nigricans* highlights the importance of better understanding the ecological interactions between bats and their parasites, even in the absence of direct evidence of harmful impacts (Schaer et al. 2013). The preservation of natural habitats, such as the Atlantic Forest, is crucial not only for biodiversity but also for maintaining these complex ecological relationships. Environmental degradation, such as deforestation, can modify the dynamics of parasite transmission by altering the interaction between bats and their vectors (Dick and Patterson 2006; Gottdenker et al. 2014; Keesing et al. 2010). Thus, the conservation of preserved areas becomes essential to ensure the stability of these interactions and to protect both bat populations and the ecosystems in which they are embedded.

Although this study has made progress in the molecular detection of *Polychromophilus*, there is an urgent need for further investigations to better understand the ecology of these parasite–vector–host interactions. Future studies should include sampling in various regions of Brazil, encompassing different biomes, bats, and vectors, to assess the distribution and genetic diversity of *Polychromophilus*. Furthermore, it is important to investigate the pathogenicity of the parasites in bats and the transmission dynamics in varied environments.

## Conflicts of Interest

The authors declare no conflicts of interest.

## Supporting information




**Table S1** Mitochondrial cytochrome b (*cytb*) gene sequences used in phylogenetic analyzes and their respective GenBank accession numbers
**Table S2** Information on each individual specimen and locality with respective geographic coordinates
**Table S3** Similarity percentage between the mitochondrial cytochrome b gene (*cytb*) sequences of *Polychromophilus* sp. found in different hosts from Brazil, Panama and Colombia
**Table S4** Mitochondrial gene cytochrome b (*cytb*), nuclear gene adenylosuccinate lyase (*asl*) and apicoplast gene caseinolytic protease C (*clpc*) sequences from *Polychromophilus* species used in phylogenetic analyzes and their respective GenBank accession numbers
