# Supplementary material for: Polychromophilus spp. (Haemosporida: Plasmodiidae): First Molecular Detection in Bat Flies From Brazilian Bats
Source: Integr Zool. 2025 May 29;21(1):137–50. doi: 10.1111/1749-4877.13001 (PMC12794751; doi:10.1111/1749-4877.13001)
Supplement: Supplementary file 1 — Table S1 Mitochondrial cytochrome b (cytb) gene sequences used in phylogenetic analyzes and their respective GenBank accession numbers Table S2 Information on each individual specimen and locality with respective geographic coordinates Table S3 Similarity percentage between the mitochondrial cytochrome b gene (cytb) sequences of Polychromophilus sp. found in different hosts from Brazil, Panama and Colombia Table S4 Mitochondrial gene cytochrome b (cytb), nuclear gene adenylosuccinate lyase (asl) and apicoplast gene caseinolytic protease C (clpc) sequences from Polychromophilus species used in phylogenetic analyzes and their respective GenBank accession numbers [file INZ2-21-137-s001.doc]

**SUPPLEMENTARY MATERIALS**

**Table S1** Mitochondrial cytochrome b (*cytb*) gene sequences used in phylogenetic analyzes and their respective GenBank® accession numbers. Sequences from this study are highlighted in bold.

| **GenBank®**  **Accession Number** | **Parasite**  **Species** | **Host**  **Species** | **Country of Source** |
| --- | --- | --- | --- |
| MN316537, MN316538 | *Haemocystidium cf. chelodinae* | *Myuchelys georgesi* | Australia |
| MK976708-MK976710 | *Haemocystidium pacayae* | *Podocnemis vogli* | Colombia |
| MH177855 | *Haemocystidium ptyodactylii* | Squamate * | unknown |
| KT364883 | *Haemocystidium* sp. | *Hemidactylus luqueorum* | Oman |
| KT364884 | *Haemocystidium* sp. | *Ptyodactylus hasselquistii* | Oman |
| KX148083-KX148085 | *Haemocystidium* sp. | *Kinixys erosa* | Gabon |
| KX148088-KX148090 | *Haemocystidium* sp. | *Kinixys erosa* | Gabon |
| KX148086, KX148087 | *Haemocystidium* sp. | *Pelusios castaneus* | Gabon |
| MT684458 | *Haemocystidium* sp. | *Podocnemis vogli* | Colombia |
| MT684459 | *Haemocystidium* sp. | *Trachylepis spilogaster* | Angola |
| MT684460 | *Haemocystidium* sp. | *Rhacodactylus auriculatus* | New Caledonia |
| DQ630007 | *Haemoproteus balmorali* | *Luscinia luscina* | Lithuania |
| DQ630014 | *Haemoproteus balmorali* | *Muscicapa striata* | Lithuania |
| DQ630006 | *Haemoproteus belopolskyi* | *Hippolais icterina* | Sweden |
| MK843310 | *Haemoproteus belopolskyi* | *Hippolais icterina* | Lithuania |
| FJ168562 | *Haemoproteus columbae* | *Columba livia* | USA |
| MK843311 | *Haemoproteus hirundinis* | *Delichon urbicum* | Lithuania |
| KY653778 | *Haemoproteus iwa* | *Fregata magnificens* | Ecuador |
| KY653760 | *Haemoproteus jenniae* | *Creagrus furcatus* | Ecuador |
| DQ630010 | *Haemoproteus lanii* | *Lanius collurio* | Russia |
| MK843313 | *Haemoproteus lanii* | *Lanius collurio* | Lithuania |
| AY099045 | *Haemoproteus majoris* | *Parus caeruleus* | Sweden |
| JN164727, JN164728 | *Haemoproteus majoris* | *Sylvia atricapilla* | Spain |
| KU160476 | *Haemoproteus minchini* | *Corythaeola cristata* | Singapore |
| DQ630013 | *Haemoproteus minutus* | *Turdus merula* | Lithuania |
| KY653756 | *Haemoproteus multipigmentatus* | *Zenaida galapagoensis* | Ecuador |
| MK843312 | *Haemoproteus nucleocondensus* | *Acrocephalus arundinaceus* | Lithuania |
| JN164720 | *Haemoproteus pallidulus* | *Sylvia atricapilla* | Spain |
| DQ630004, DQ630005 | *Haemoproteus pallidus* | *Ficedula hypoleuca* | Sweden, Russia |
| JN164718, JN164719, JN164722 | *Haemoproteus parabelopolskyi* | *Sylvia atricapilla* | Spain |
| DQ630009 | *Haemoproteus payevsky* | *Acrocephalus scipaceus* | Lithuania |
| AY099040 | *Haemoproteus sylvae* | *Acrocephalus arundinaceus* | Sweden |
| OP503501 | *Haemosporida* sp. | *Noctilio albiventris* | Brazil |
| FJ168565 | *Hepatocystis* sp. | *Pteropus hypomelanus* | USA |
| JQ070951, JQ070956 | *Hepatocystis* sp. | *Cercopithecus nictitans* | Cameroon |
| FJ168563 | *Leucocytozoon majoris* | *Zonotrichia leucophrys oriantha* | USA |
| NC_012450 | *Leucocytozoon majoris* | *Zonotrichia leucophrys oriantha* | USA |
| KF159690 | *Nycteria* sp. | *Rhinolophus landeri* | Guinea |
| KF159720 | *Nycteria* sp. | *Rhinolophus alcyone* | Côte d’Ivoire |
| MK098843-MK098847 | *Nycteria* sp. | *Rhinolophus* sp.*, R. landeri* | Gabon |
| FJ168561 | *Parahaemoproteus vireonis* | *Vireo gilvus* | USA |
| NC_012447 | *Parahaemoproteus vireonis* | *Vireo gilvus* | USA |
| HQ712051 | *Plasmodium atheruri* | *Atherurus africanus* | Madagascar |
| AY099055 | *Plasmodium azurophilum* | *Anolis oculatus* | Dominica |
| AY377128 | *Plasmodium cathemerium* | *Serinus canaria* | Germany |
| JN164734 | *Plasmodium circumflexum* | *Sylvia atricapilla* | Spain |
| AB444126 | *Plasmodium cynomolgi* | Monkey * | Japan |
| AF069611 | *Plasmodium elongatum* | *Passer domesticus* | North America |
| JF923762 | *Plasmodium falciparum* | *Cercopithecus nictitans* | Gabon |
| FJ895307 | *Plasmodium gaboni* | *Pan* sp. | Gabon |
| AY099053 | *Plasmodium giganteum* | *Agama agama* | Ghana |
| JF923751 | *Plasmodium gonderi* | *Mandrillus sphinx* | Gabon |
| JQ345504 | *Plasmodium knowlesi* | *Homo sapiens* | Malaysia |
| HM000110 | *Plasmodium malariae* | *Pan troglodytes ellioti* | Cameroon |
| OP503500 | *Plasmodium malariae* | *Homo sapiens* | Brazil |
| GU723548 | *Plasmodium ovale* | *Homo sapiens* | England |
| AY733090 | *Plasmodium relictum* | *Hemignathus virens* | USA |
| HM222485 | *Plasmodium* sp. | *Icteria virens* | USA |
| HM235065 | *Plasmodium* sp. | *Gorilla* sp. | Cameroon |
| HM235081 | *Plasmodium* sp. | *Gorilla* sp. | Cameroon |
| KF591834 | *Plasmodium vivax* | *Homo sapiens* | Congo |
| DQ414658 | *Plasmodium yoelii killicki* | *Thamnomys rutilans* | Congo |
| JN990708-JN990711 | *Polychromophilus melanipherus* | *Miniopterus schreibersii* | Switzerland |
| KJ131270-KJ131275 | *Polychromophilus melanipherus* | *Miniopterus schreibersii* | Europa |
| KU182361-KU182367 | *Polychromophilus melanipherus* | *Nycteribia schmidlii scotti* | Gabon |
| KU182368 | *Polychromophilus melanipherus* | *Penicillidia fulvida* | Gabon |
| MH744504, MH744505 | *Polychromophilus melanipherus* | *Miniopterus mahafaliensis* | Madagascar |
| MH744506, MH744519 | *Polychromophilus melanipherus* | *Miniopterus griffithsi* | Madagascar |
| MH744508 | *Polychromophilus melanipherus* | *Miniopterus griveaudi* | Madagascar |
| MH744522-MH744525 | *Polychromophilus melanipherus* | *Miniopterus griveaudi* | Madagascar |
| MH744509-MH744511 | *Polychromophilus melanipherus* | *Miniopterus gleni* | Madagascar |
| MH744518, MH744521 | *Polychromophilus melanipherus* | *Miniopterus gleni* | Madagascar |
| MH744512, MH744526 | *Polychromophilus melanipherus* | *Miniopterus manavi* | Madagascar |
| MH744514-MH744516 | *Polychromophilus melanipherus* | *Miniopterus griveaudi* | Madagascar |
| MH744520 | *Polychromophilus melanipherus* | *Paratriaenops furculus* | Madagascar |
| MH744527 | *Polychromophilus melanipherus* | *Nycteribia stylidiopsis* | Madagascar |
| MH744528-MH744531 | *Polychromophilus melanipherus* | *Penicillidia leptothrinax* | Madagascar |
| MK088162-MK088164, MK088168 | *Polychromophilus melanipherus* | *Miniopterus orianae* | Australia |
| MT136167 | *Polychromophilus melanipherus* | *Taphozous melanopogon* | Thailand |
| MW007671-MW007674 | *Polychromophilus melanipherus* | *Nycteribia schmidlii scotti* | South Africa |
| MW007676 | *Polychromophilus melanipherus* | *Nycteribia schmidlii scotti* | South Africa |
| MW007677 | *Polychromophilus melanipherus* | *Miniopterus natalensis* | South Africa |
| MW007680-MW007682 | *Polychromophilus melanipherus* | *Nycteribia schmidlii* | Hungary |
| MW007685 | *Polychromophilus melanipherus* | *Nycteribia schmidlii* | Spain |
| MW007689 | *Polychromophilus melanipherus* | *Miniopterus schreibersii* | Spain |
| HM055583 | *Polychromophilus murinus* | *Myotis daubentonii* | Switzerland |
| HM055583 | *Polychromophilus murinus* | *Eptesicus serotinus* | Switzerland |
| HM055583 | *Polychromophilus murinus* | *Nyctalus noctula* | Switzerland |
| HM055583 | *Polychromophilus murinus* | *Myotis myotis* | Switzerland |
| HM055584-HM055589 | *Polychromophilus murinus* | *Myotis daubentonii* | Switzerland |
| JN990712, JN990713 | *Polychromophilus murinus* | *Myotis daubentonii* | Switzerland |
| MH744532-MH744536 | *Polychromophilus murinus* | *Myotis goudoti* | Madagascar |
| MH744537 | *Polychromophilus murinus* | *Penicillidia* sp. | Madagascar |
| MT136168 | *Polychromophilus murinus* | *Myotis siligorensis* | Thailand |
| KF159675, KF159681 | *Polychromophilus* sp. | *Miniopterus villiersi* | Guinea |
| KF159699 | *Polychromophilus* sp. | *Miniopterus villiersi* | Guinea |
| KF159700 | *Polychromophilus* sp*.* | *Neoromicia capensis* | Guinea |
| LN483036 | *Polychromophilus* sp. | *Rhinolophus sp.* | Bulgaria |
| LN483038 | *Polychromophilus* sp. | *Myotis nigricans* | Panama |
| MK098848, MK098849 | *Polychromophilus* sp. | *Miniopterus minor* | Gabon |
| OP503502 | *Polychromophilus* sp. | *Myotis riparius* | Brazil |
| JQ995284-JQ995288 | *Polychromophilus* sp. | *Miniopterus inflatus* | Gabon |
| KF159714 | *Polychromophilus* sp. | *Pipistrellus aff. grandidieri* | Guinea |
| MT750305, MT750307, MT750308 | *Polychromophilus* sp. | *Scotophilus kuhlii* | Thailand |
| LC668431 | *Polychromophilus murinus* | *Myotis macrodactylus* | Japan |
| LC668432 | *Polychromophilus murinus* | *Myotis macrodactylus* | Japan |
| LC668433 | *Polychromophilus murinus* | *Myotis macrodactylus* | Japan |
| MW984518 | *Polychromophilus* sp. | *Myotis ruber* | Brazil |
| MW984519, MW984520 | *Polychromophilus* sp. | *Myotis riparius* | Brazil |
| MW984522 | *Polychromophilus* sp. | *Myotis riparius* | Brazil |
| MW984521 | *Polychromophilus* sp. | *Neoeptesicus diminutus* | Brazil |
| OQ957064 | *Polychromophilus* sp. | *Myotis ruber* | Brazil |
| OQ957065 | *Polychromophilus* sp. | *Myotis* sp. | Brazil |
| OQ957066 | *Polychromophilus* sp. | *Myotis* sp. | Brazil |
| PP971136 | *Polychromophilus deanei* | *Myotis albescens* | Colombia |
| **PQ789623** | *Polychromophilus* sp. | *Basilia lindolphoi* (ID 16_2) | Brazil |
| **PQ789624** | *Polychromophilus* sp. | *Basilia speiseri* (ID 85_1) | Brazil |
| * unreported species. |  |  |  |

**Table S2** Information on each individual specimen and locality with respective geographic coordinates.

| **Sample ID** | **Host Family** | **Host Bats** | **Bat fly Family** | **Bat fly species** | **Sexing** | **Coordenates** | **Locality** |
| --- | --- | --- | --- | --- | --- | --- | --- |
| 05_1 | Verpertilionidae | *Myotis nigricans* | Nycteribiidae | *Basilia speiseri* | F | 24° 1'49.73"S 47°21'36.53"W | Tapiraí-SP |
| 05_2 | Verpertilionidae | *Myotis nigricans* | Nycteribiidae | *Basilia speiseri* | F | 24° 1'49.73"S 47°21'36.53"W | Tapiraí-SP |
| 05_3 | Verpertilionidae | *Myotis nigricans* | Nycteribiidae | *Basilia speiseri* | F | 24° 1'49.73"S 47°21'36.53"W | Tapiraí-SP |
| 05_4 | Verpertilionidae | *Myotis nigricans* | Nycteribiidae | *Basilia speiseri* | F | 24° 1'49.73"S 47°21'36.53"W | Tapiraí-SP |
| 05_5 | Verpertilionidae | *Myotis nigricans* | Nycteribiidae | *Basilia speiseri* | F | 24° 1'49.73"S 47°21'36.53"W | Tapiraí-SP |
| 12_1 | Verpertilionidae | *Myotis nigricans* | Nycteribiidae | *Basilia speiseri* | F | 24° 1'49.73"S 47°21'36.53"W | Tapiraí-SP |
| 12_2 | Verpertilionidae | *Myotis nigricans* | Nycteribiidae | *Basilia speiseri* | F | 24° 1'49.73"S 47°21'36.53"W | Tapiraí-SP |
| 12_3 | Verpertilionidae | *Myotis nigricans* | Nycteribiidae | *Basilia speiseri* | F | 24° 1'49.73"S 47°21'36.53"W | Tapiraí-SP |
| 12_4 | Verpertilionidae | *Myotis nigricans* | Nycteribiidae | *Basilia speiseri* | F | 24° 1'49.73"S 47°21'36.53"W | Tapiraí-SP |
| 12_5 | Verpertilionidae | *Myotis nigricans* | Nycteribiidae | *Basilia speiseri* | F | 24° 1'49.73"S 47°21'36.53"W | Tapiraí-SP |
| 12_6 | Verpertilionidae | *Myotis nigricans* | Nycteribiidae | *Basilia speiseri* | F | 24° 1'49.73"S 47°21'36.53"W | Tapiraí-SP |
| 12_7 | Verpertilionidae | *Myotis nigricans* | Nycteribiidae | *Basilia speiseri* | F | 24° 1'49.73"S 47°21'36.53"W | Tapiraí-SP |
| 12_8 | Verpertilionidae | *Myotis nigricans* | Nycteribiidae | *Basilia speiseri* | M | 24° 1'49.73"S 47°21'36.53"W | Tapiraí-SP |
| 16_2 | Verpertilionidae | *Myotis nigricans* | Nycteribiidae | ***Basilia lindolphoi*** | M | 24° 1'49.73"S 47°21'36.53"W | Tapiraí-SP |
| 20_1 | Phyllostomidae | *Anoura caudifer* | Streblidae | *Strebla carvalhoi* | F | 24° 3'19.47"S 47°26'12.86"W | Tapiraí-SP |
| 20_2 | Phyllostomidae | *Anoura caudifer* | Streblidae | *Anastrebla caudiferae* | M | 24° 3'19.47"S 47°26'12.86"W | Tapiraí-SP |
| 20_3 | Phyllostomidae | *Anoura caudifer* | Streblidae | *Anastrebla caudiferae* | F | 24° 3'19.47"S 47°26'12.86"W | Tapiraí-SP |
| 20_4 | Phyllostomidae | *Anoura caudifer* | Streblidae | *Anastrebla caudiferae* | M | 24° 3'19.47"S 47°26'12.86"W | Tapiraí-SP |
| 22_1 | Verpertilionidae | *Neoeptesicus* sp. | Nycteribiidae | *Basilia* sp. | M | 24°05'04.7"S 47°28'37.4"W | Miracatu-SP |
| 22_2 | Verpertilionidae | *Neoeptesicus* sp. | Nycteribiidae | *Basilia* sp. | M | 24°05'04.7"S 47°28'37.4"W | Miracatu-SP |
| 29 | Phyllostomidae | *Carollia perspicillata* | Streblidae | *Trichobius joblingi* | F | 24° 1'49.73"S 47°21'36.53"W | Tapiraí-SP |
| 33_1 | Phyllostomidae | *Carollia perspicillata* | Streblidae | *Paraeuctenodes similis* | F | 24° 1'49.73"S 47°21'36.53"W | Tapiraí-SP |
| 33_2 | Phyllostomidae | *Carollia perspicillata* | Streblidae | *Paraeuctenodes similis* | M | 24° 1'49.73"S 47°21'36.53"W | Tapiraí-SP |
| 33_3 | Phyllostomidae | *Carollia perspicillata* | Streblidae | *Strebla guajiro* | F | 24° 1'49.73"S 47°21'36.53"W | Tapiraí-SP |
| 33_4 | Phyllostomidae | *Carollia perspicillata* | Streblidae | *Trichobius joblingi* | F | 24° 1'49.73"S 47°21'36.53"W | Tapiraí-SP |
| 33_5 | Phyllostomidae | *Carollia perspicillata* | Streblidae | *Trichobius joblingi* | M | 24° 1'49.73"S 47°21'36.53"W | Tapiraí-SP |
| 33_6 | Phyllostomidae | *Carollia perspicillata* | Streblidae | *Trichobius joblingi* | M | 24° 1'49.73"S 47°21'36.53"W | Tapiraí-SP |
| 41_1 | Phyllostomidae | *Sturnira lilium* | Streblidae | *Aspidoptera falcata* | F | 24° 1'49.73"S 47°21'36.53"W | Tapiraí-SP |
| 41_2 | Phyllostomidae | *Sturnira lilium* | Streblidae | *Aspidoptera falcata* | M | 24° 1'49.73"S 47°21'36.53"W | Tapiraí-SP |
| 41_3 | Phyllostomidae | *Sturnira lilium* | Streblidae | *Aspidoptera falcata* | M | 24° 1'49.73"S 47°21'36.53"W | Tapiraí-SP |
| 41_4 | Phyllostomidae | *Sturnira lilium* | Streblidae | *Aspidoptera falcata* | M | 24° 1'49.73"S 47°21'36.53"W | Tapiraí-SP |
| 58_1 | Phyllostomidae | *Carollia perspicillata* | Streblidae | *Trichobius joblingi* | M | 24° 1'49.73"S 47°21'36.53"W | Tapiraí-SP |
| 58_2 | Phyllostomidae | *Carollia perspicillata* | Streblidae | *Trichobius joblingi* | M | 24° 1'49.73"S 47°21'36.53"W | Tapiraí-SP |
| 58_3 | Phyllostomidae | *Carollia perspicillata* | Streblidae | *Trichobius joblingi* | M | 24° 1'49.73"S 47°21'36.53"W | Tapiraí-SP |
| 58_4 | Phyllostomidae | *Carollia perspicillata* | Streblidae | *Trichobius joblingi* | M | 24° 1'49.73"S 47°21'36.53"W | Tapiraí-SP |
| 59_1 | Phyllostomidae | *Anoura caudifer* | Streblidae | *Trichobius tiptoni* | M | 24° 1'49.73"S 47°21'36.53"W | Tapiraí-SP |
| 59_2 | Phyllostomidae | *Anoura caudifer* | Streblidae | *Trichobius tiptoni* | M | 24° 1'49.73"S 47°21'36.53"W | Tapiraí-SP |
| 60 | Phyllostomidae | *Anoura caudifer* | Streblidae | *Trichobius tiptoni* | F | 24° 1'49.73"S 47°21'36.53"W | Tapiraí-SP |
| 62 | Verpertilionidae | *Myotis nigricans* | Nycteribiidae | *Basilia speiseri* | M | 24°05'04.7"S 47°28'37.4"W | Miracatu-SP |
| 63_1 | Verpertilionidae | *Myotis nigricans* | Nycteribiidae | *Basilia speiseri* | M | 24°05'04.7"S 47°28'37.4"W | Miracatu-SP |
| 63_2 | Verpertilionidae | *Myotis nigricans* | Nycteribiidae | *Basilia speiseri* | F | 24°05'04.7"S 47°28'37.4"W | Miracatu-SP |
| 65_1 | Verpertilionidae | *Myotis nigricans* | Nycteribiidae | *Basilia speiseri* | M | 24°05'04.7"S 47°28'37.4"W | Miracatu-SP |
| 65_2 | Verpertilionidae | *Myotis nigricans* | Nycteribiidae | *Basilia speiseri* | F | 24°05'04.7"S 47°28'37.4"W | Miracatu-SP |
| 66_1 | Phyllostomidae | *Glossophaga soricina* | Streblidae | *Trichobius dugesii* | M | 24°05'04.7"S 47°28'37.4"W | Miracatu-SP |
| 66_2 | Phyllostomidae | *Glossophaga soricina* | Streblidae | *Trichobius tiptoni* | M | 24°05'04.7"S 47°28'37.4"W | Miracatu-SP |
| 84 | Verpertilionidae | *Myotis nigricans* | Nycteribiidae | *Basilia speiseri* | M | 24°05'04.7"S 47°28'37.4"W | Miracatu-SP |
| 85_1 | Verpertilionidae | *Myotis nigricans* | Nycteribiidae | ***Basilia speiseri*** | F | 24°05'04.7"S 47°28'37.4"W | Miracatu-SP |
| 85_2 | Verpertilionidae | *Myotis nigricans* | Nycteribiidae | *Basilia speiseri* | F | 24°05'04.7"S 47°28'37.4"W | Miracatu-SP |
| 116_1 | Phyllostomidae | *Carollia perspicillata* | Streblidae | *Megistopoda proxima* | M | 24° 1'49.73"S 47°21'36.53"W | Tapiraí-SP |
| 116_2 | Phyllostomidae | *Carollia perspicillata* | Streblidae | *Megistopoda proxima* | M | 24° 1'49.73"S 47°21'36.53"W | Tapiraí-SP |
| 121_1 | Phyllostomidae | *Sturnira lilium* | Streblidae | *Aspidoptera falcata* | F | 24° 3'19.47"S 47°26'12.86"W | Tapiraí-SP |
| 121_2 | Phyllostomidae | *Sturnira lilium* | Streblidae | *Megistopoda proxima* | M | 24° 3'19.47"S 47°26'12.86"W | Tapiraí-SP |
| 121_3 | Phyllostomidae | *Sturnira lilium* | Streblidae | *Aspidoptera falcata* | F | 24° 3'19.47"S 47°26'12.86"W | Tapiraí-SP |
| 121_4 | Phyllostomidae | *Sturnira lilium* | Streblidae | *Aspidoptera falcata* | M | 24° 3'19.47"S 47°26'12.86"W | Tapiraí-SP |
| 121_5 | Phyllostomidae | *Sturnira lilium* | Streblidae | *Megistopoda proxima* | M | 24° 3'19.47"S 47°26'12.86"W | Tapiraí-SP |
| 122_1 | Phyllostomidae | *Carollia perspicillata* | Streblidae | *Strebla guajiro* | M | 24° 3'19.47"S 47°26'12.86"W | Tapiraí-SP |
| 122_2 | Phyllostomidae | *Carollia perspicillata* | Streblidae | *Trichobius joblingi* | M | 24° 3'19.47"S 47°26'12.86"W | Tapiraí-SP |
| 122_3 | Phyllostomidae | *Carollia perspicillata* | Streblidae | *Trichobius joblingi* | F | 24° 3'19.47"S 47°26'12.86"W | Tapiraí-SP |
| 123 | Phyllostomidae | *Sturnira lilium* | Streblidae | *Megistopoda proxima* | M | 24° 3'19.47"S 47°26'12.86"W | Tapiraí-SP |
| 127 | Phyllostomidae | *Sturnira lilium* | Streblidae | *Megistopoda proxima* | M | 24° 3'19.47"S 47°26'12.86"W | Tapiraí-SP |
| 130_1 | Phyllostomidae | *Anoura caudifer* | Streblidae | *Trichobius tiptoni* | F | 24° 3'19.47"S 47°26'12.86"W | Tapiraí-SP |
| 130_2 | Phyllostomidae | *Anoura caudifer* | Streblidae | *Trichobius tiptoni* | F | 24° 3'19.47"S 47°26'12.86"W | Tapiraí-SP |
| 134_1 | Phyllostomidae | *Carollia perspicillata* | Streblidae | *Strebla guajiro* | F | 24° 3'19.47"S 47°26'12.86"W | Tapiraí-SP |
| 134_2 | Phyllostomidae | *Carollia perspicillata* | Streblidae | *Trichobius joblingi* | F | 24° 3'19.47"S 47°26'12.86"W | Tapiraí-SP |
| 134_3 | Phyllostomidae | *Carollia perspicillata* | Streblidae | *Trichobius joblingi* | M | 24° 3'19.47"S 47°26'12.86"W | Tapiraí-SP |
| 134_4 | Phyllostomidae | *Carollia perspicillata* | Streblidae | *Trichobius joblingi* | F | 24° 3'19.47"S 47°26'12.86"W | Tapiraí-SP |
| 137 | Phyllostomidae | *Desmodus rotundus* | Streblidae | *Strebla wiedemanni* | M | 24° 3'19.47"S 47°26'12.86"W | Tapiraí-SP |

**In bold, specimens positive for *Polychromophilus* sp.**

**Table S3** Similarity percentage between the mitochondrial cytochrome b gene (*cytb*) sequences of *Polychromophilus* sp. found in different hosts from Brazil, Panama and Colombia.

| **Sample ID** | **116** | **198** | **335** | **607** | **650** | **69642** | **125** | **138** | **141** | **16_2** | **85_1** | **PP971136** | **MYOPA01** |
| --- | --- | --- | --- | --- | --- | --- | --- | --- | --- | --- | --- | --- | --- |
| **MW984518** | 1116 | 99% | 99% | 99% | 99% | 99% | 99% | 99% | 99% | 99% | 99% | 100% | 99% |
| **MW984519** | 1113 | 1116 | 100% | 99% | 99% | 99% | 99% | 99% | 99% | 99% | 99% | 99% | 99% |
| **MW984520** | 1113 | 1116 | 1116 | 99% | 99% | 99% | 99% | 99% | 99% | 99% | 99% | 99% | 99% |
| **OP503502** | 1115 | 1112 | 1112 | 1116 | 98% | 99% | 98% | 99% | 99% | 99% | 99% | 99% | 99% |
| **MW984521** | 1105 | 1105 | 1105 | 1104 | 1116 | 98% | 99% | 98% | 99% | 98% | 98% | 99% | 98% |
| **MW984522** | 1115 | 1112 | 1112 | 1114 | 1104 | 1116 | 98% | 99% | 99% | 99% | 99% | 100% | 99% |
| **OQ957064** | 1105 | 1105 | 1105 | 1104 | 1113 | 1104 | 1116 | 98% | 99% | 98% | 98% | 99% | 98% |
| **OQ957065** | 1111 | 1109 | 1109 | 1110 | 1102 | 1110 | 1102 | 1116 | 99% | 99% | 99% | 99% | 98% |
| **OQ957066** | 1115 | 1113 | 1113 | 1114 | 1106 | 1114 | 1106 | 1112 | 1116 | 99% | 99% | 99% | 99% |
| **PQ789623*** | 1115 | 1112 | 1112 | 1114 | 1104 | 1114 | 1104 | 1110 | 1114 | 1116 | 99% | 99% | 99% |
| **PQ789624*** | 1110 | 1108 | 1108 | 1109 | 1101 | 1109 | 1101 | 1113 | 1111 | 1109 | 1116 | 99% | 98% |
| **PP971136** | 498 | 497 | 497 | 497 | 494 | 498 | 493 | 494 | 497 | 497 | 495 | 498 | 99% |
| **MYOPA01** | 592 | 591 | 591 | 591 | 587 | 592 | 586 | 588 | 591 | 591 | 586 | 352 | 595 |

*Samples this study.

**Table S4** Mitochondrial gene cytochrome b (*cytb*), nuclear gene adenylosuccinate lyase (*asl*) and apicoplast gene caseinolytic protease C (*clpc*) sequences from *Polychromophilus* species used in phylogenetic analyzes and their respective GenBank® accession numbers. Sequences from this study are highlighted in bold.

| **Host Species** | **Parasite Species** | ***cytb*** | ***asl*** | ***clpc*** | **Country of Source** |
| --- | --- | --- | --- | --- | --- |
| *Miniopterus schreibersii* | *Polychromophilus melanipherus* | JN990708 | - | JN990720 | Switzerland |
| *Miniopterus schreibersii* | *Polychromophilus melanipherus* | JN990709 | JN990726 | JN990721 | Switzerland |
| *Miniopterus schreibersii* | *Polychromophilus melanipherus* | JN990710 | - | JN990722 | Switzerland |
| *Myotis daubentonii* | *Polychromophilus murinus* | JN990712 | JN990725 | JN990723 | Switzerland |
| *Myotis daubentonii* | *Polychromophilus murinus* | JN990713 | - | JN990724 | Switzerland |
| *Miniopterus villiersi* | *Polychromophilus sp.* | KF159699 | - | KF159616 | Guinea |
| *Neoromicia capensis* | *Polychromophilus sp.* | KF159681 | - | KF159642 | Guinea |
| *Pipistrellus aff. grandidieri* | *Polychromophilus sp.* | KF159714 | - | KF159639 | Guinea |
| *Miniopterus natalensis* | *Polychromophilus melanipherus* | KT750379 | KT750646 | KT750738 | Kenya |
| *Miniopterus natalensis* | *Polychromophilus melanipherus* | KT750382 | KT750633 | - | Kenya |
| *Miniopterus natalensis* | *Polychromophilus melanipherus* | KT750380 | KT750647 | KT750740 | Kenya |
| *Miniopterus rufus* | *Polychromophilus melanipherus* | KT750385 | KT750637 | KT750745 | Kenya |
| *Miniopterus rufus* | *Polychromophilus melanipherus* | KT750386 | - | KT750748 | Kenya |
| *Miniopterus sp.* | *Polychromophilus melanipherus* | KT750387 | KT750642 | KT750749 | Kenya |
| *Miniopterus natalensis* | *Polychromophilus melanipherus* | KT750377 | KT750629 | - | Kenya |
| *Miniopterus africanus* | *Polychromophilus melanipherus* | KT750375 | KT750627 | KT750734 | Kenya |
| *Miniopterus natalensis* | *Polychromophilus melanipherus* | KT750400 | KT750630 | KT750737 | Kenya |
| *Miniopterus rufus* | *Polychromophilus melanipherus* | KT750403 | KT750636 | KT750744 | Kenya |
| *Miniopterus rufus* | *Polychromophilus melanipherus* | KT750404 | KT750639 | KT750746 | Kenya |
| *Miniopterus rufus* | *Polychromophilus melanipherus* | KT750418 | KT750641 | - | Kenya |
| *Miniopterus natalensis* | *Polychromophilus melanipherus* | KT750376 | KT750628 | KT750735 | Kenya |
| *Miniopterus natalensis* | *Polychromophilus melanipherus* | KT750401 | KT750631 | KT750739 | Kenya |
| *Miniopterus natalensis* | *Polychromophilus melanipherus* | KT750402 | KT750648 | KT750742 | Kenya |
| *Miniopterus natalensis* | *Polychromophilus melanipherus* | KT750406 | - | KT750743 | Kenya |
| *Miniopterus natalensis* | *Polychromophilus melanipherus* | KT750378 | - | KT750736 | Kenya |
| *Miniopterus minor* | *Polychromophilus sp.* | KT750388 | KT750643 | KT750750 | Tanzania |
| *Miniopterus minor* | *Polychromophilus sp.* | KT750428 | KT750644 | KT750751 | Tanzania |
| *Miniopterus minor* | *Polychromophilus sp.* | KT750429 | KT750645 | - | Tanzania |
| *Miniopterus sp.* | *Polychromophilus sp.* | KT750389 | KT750552 | KT750651 | Mozambique |
| *Miniopterus rufus* | *Polychromophilus sp.* | KT750384 | KT750635 | - | Kenya |
| *Miniopterus rufus* | *Polychromophilus sp.* | KT750383 | KT750634 | - | Kenya |
| *Miniopterus natalensis* | *Polychromophilus sp.* | KT750381 | KT750632 | KT750741 | Kenya |
| *Miniopterus rufus* | *Polychromophilus sp.* | KT750412 | KT750638 | - | Kenya |
| *Miniopterus rufus* | *Polychromophilus sp.* | KT750405 | KT750640 | KT750747 | Kenya |
| *Scotophilus kuhlii* | *Polychromophilus sp.* | MT750307 | - | MT750315 | Thailand |
| *Myotis macrodactylus* | *Polychromophilus murinus* | LC668431 | - | LC715204 | Japan |
| *Myotis macrodactylus* | *Polychromophilus murinus* | LC668432 | - | LC715203 | Japan |
| *Myotis macrodactylus* | *Polychromophilus murinus* | LC668433 | - | LC715205 | Japan |
| *Myotis riparius* | *Polychromophilus sp.* | MW984519 | - | OP503503 | Brazil |
| *Myotis riparius* | *Polychromophilus sp.* | OP503502 | - | OP503504 | Brazil |
| *Myotis ruber* | *Polychromophilus sp.* | OQ957064 | OQ957067 | - | Brazil |
| *Myotis sp.* | *Polychromophilus sp.* | OQ957066 | OQ957068 | OQ957063 | Brazil |
| *Basilia lindolphoi* (ID 16_2) | *Polychromophilus sp.* | **PQ789623** | - | **PQ789625** | Brazil |
| *Basilia speiseri* (ID 85_1) | *Polychromophilus sp.* | **PQ789624** | **PQ789627** | **PQ789626** | Brazil |

(-) represents missing data.
